# Handheld Lung Ultrasound to Detect COVID-19 Pneumonia in Inpatients: A Prospective Cohort Study

**DOI:** 10.24908/pocus.v8i2.16484

**Published:** 2023-11-27

**Authors:** Thomas F Heyne, Kay Negishi, Daniel S Choi, Ahad A Al Saud, Lucas X Marinacci, Patrick Y Smithedajkul, Lily R Devaraj, Brent P Little, Dexter P Mendoza, Efren J Flores, Milena Petranovic, Steven P Toal, Hamid Shokoohi, Andrew S Liteplo, Benjamin P Geisler

**Affiliations:** 1 Department of Medicine, Massachusetts General Hospital Boston, MA USA; 2 Department of Pediatrics, Massachusetts General Hospital Boston, MA USA; 3 Department of Emergency Medicine, Massachusetts General Hospital Boston, MA USA; 4 Department of Emergency Medicine, King Saud University College of Medicine Riyadh Saudi Arabia; 5 Richard A. and Susan F. Smith Center for Outcomes Research, Beth Israel Deaconess Medical Center Boston, MA USA; 6 Department of Radiology, Massachusetts General Hospital Boston, MA USA; 7 Institute for Medical Information Processing, Biometry, and Epidemiology, Ludwig Maximilian University Munich Germany

**Keywords:** Internal Medicine, ultra-portable, tomography, Point of Care Ultrasound (POCUS), handheld ultrasound, COVID-19

## Abstract

**Background**: Chest imaging, including chest X-ray (CXR) and computed tomography (CT), can be a helpful adjunct to nucleic acid test (NAT) in the diagnosis and management of Coronavirus Disease 2019 (COVID-19). Lung point of care ultrasound (POCUS), particularly with handheld devices, is an imaging alternative that is rapid, highly portable, and more accessible in low-resource settings. A standardized POCUS scanning protocol has been proposed to assess the severity of COVID-19 pneumonia, but it has not been sufficiently validated to assess diagnostic accuracy for COVID-19 pneumonia. **Purpose**: To assess the diagnostic performance of a standardized lung POCUS protocol using a handheld POCUS device to detect patients with either a positive NAT or a COVID-19-typical pattern on CT scan. Methods: Adult inpatients with confirmed or suspected COVID-19 and a recent CT were recruited from April to July 2020. Twelve lung zones were scanned with a handheld POCUS machine. Images were reviewed independently by blinded experts and scored according to the proposed protocol. Patients were divided into low, intermediate, and high suspicion based on their POCUS score. **Results**: Of 79 subjects, 26.6% had a positive NAT and 31.6% had a typical CT pattern. The receiver operator curve for POCUS had an area under the curve (AUC) of 0.787 for positive NAT and 0.820 for a typical CT. Using a two-point cutoff system, POCUS had a sensitivity of 0.90 and 1.00 compared to NAT and typical CT pattern, respectively, at the lower cutoff; it had a specificity of 0.90 and 0.89 compared to NAT and typical CT pattern at the higher cutoff, respectively. **Conclusions**: The proposed lung POCUS protocol with a handheld device showed reasonable diagnostic performance to detect inpatients with a positive NAT or typical CT pattern for COVID-19. Particularly in low-resource settings, POCUS with handheld devices may serve as a helpful adjunct for persons under investigation for COVID-19 pneumonia.

## Background

Even as newer viral variants have proven less deadly than the initial waves, Coronavirus Disease 2019 (COVID-19) continues to affect our world. Although critical to mitigate the spread of disease, rapid and accurate diagnosis of COVID-19 can be challenging. The current gold standard test to detect Severe Acute Respiratory Syndrome Coronavirus-2 (SARS-CoV-2) infection is a nucleic acid amplification test (NAT) via reverse-transcription polymerase chain reaction. NAT has excellent specificity but variable sensitivity (70-97%) 1. Indeed, a not insignificant number of patients (perhaps 2-3.5%) may be infected even with an initial negative NAT 2, 3, 4. Additionally, NAT may be expensive and require hours to result. Rapid antigen testing provides results in minutes but has lower sensitivity 5. Computed tomography (CT) of the chest, which can detect typical patterns of lung findings for COVID-19, has good sensitivity (86-97%) but lower specificity (25-81%) 6, 7, 8, 9. In some locations, CT scan is used as a diagnostic adjunct, e.g., for patients with a pending NAT test or an initial negative NAT but high clinical concern for COVID-19 10, 11. Both the World Health Organization and the Fleischner Society have recommended chest imaging for patients with moderate or severe symptoms of suspected COVID-19, even if NAT is negative 12, 13. Several validated reporting systems are used by radiologists in assessing likelihood of COVID-19 pneumonia on chest CT. A commonly used consensus guideline from the Radiological Society of North America (RSNA) classifies CT morphologies as “typical” (highest suspicion), “indeterminate”, “atypical”, or “negative” for COVID-19 pneumonia 14. For example, typical CT findings include peripheral bilateral ground-glass opacities 15. Despite its utility, CT scan has drawbacks; it requires significant healthcare resources and time, exposes the patient to ionizing radiation, poses infection control risks, and may be unsafe for unstable, hypoxemic patients. Chest X-ray (CXR) reduces some of these drawbacks but has a low sensitivity for detecting COVID-19, particularly early in the disease course (55-83%) 16. Thus, some hospitals rely on more than one category of diagnostic test, as well as patient history and risk factors, to attempt to rule out COVID-19 infection and decide on isolation precautions. In our quaternary hospital system, both CXR and CT are used in an evidence-based algorithm for admitted persons under investigation (PUIs); for example, CT is often required to allow a clinician to “rule out” COVID-19 (and remove a patient from isolation) if initial CXR findings are concerning 11. Thus, in daily clinical practice, CXR and CT are sometimes used as helpful adjuncts to help rule out and rule in COVID-19 8, 17.

**Figure 1:**
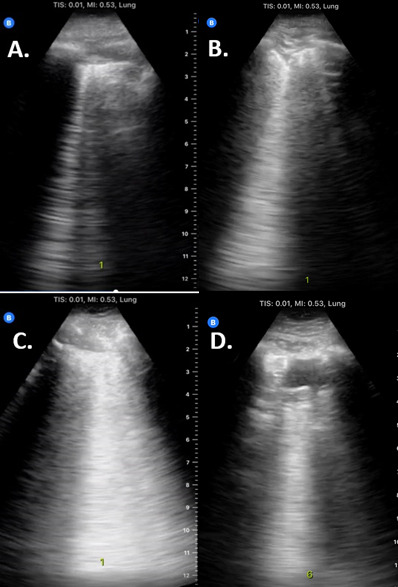
Examples of Lung Pathology. A) 1-2 B-lines. B) Broken pleural line/small subpleural consolidation. C) Confluent B-lines (light-beam artifact). D) Large subpleural consolidation. In the proposed protocol, these findings merit 1 point (A), 2 points (B), and 3 points (C or D).

Point of care ultrasound (POCUS) has been increasingly studied as a viable alternative diagnostic modality. POCUS is inexpensive, rapid, ionizing radiation-free, widely available, and does not require travel to and possible infectious exposure of a radiology suite. Ultraportable, handheld machines are typically less expensive and easier to disinfect than traditional, larger ultrasound machines. In low-resource settings, POCUS may be available when CXR or CT are not. Importantly, COVID-19 tends to affect the lung periphery, which is visualized well by POCUS. Numerous studies have described characteristic features for COVID-19 on lung ultrasound, including bilateral B-lines (particularly “confluent” B-lines) and an irregular (or “serrated”) pleural line. 15, 18, 19, 20. B-lines, similar in appearance to movie premier searchlights, appear as hyperechoic laser-like reverberation artifacts that arise from the pleural line, obliterate A-lines, and extend to the bottom of the screen (Figure 1). They can coalesce into “confluent” B-lines, which appear as a broader beam of light (appearing less as a laser and more as a broad flashlight in the fog); this artifact has also been called “light beam artifact,” “waterfall” B-line, or “white lung.” Compared to a normal pleural line, which is distinct and unbroken (something that could be drawn in one stroke by an imaginary white pencil), an irregular or “serrated” pleural line appears jagged and/or broken. Of note, while these ultrasound findings raise suspicion for COVID-19 pneumonia, they are not specific and can be seen in other pulmonary disease processes such as acute respiratory distress syndrome, cardiogenic pulmonary edema, interstitial lung disease, or pneumonia from other microbial etiologies. Of course, some CXR or CT findings suggestive of COVID-19 are similarly non-specific.

Therefore, different stratifying scoring systems to assess COVID-19 disease severity have been proposed. A widely-cited article from Soldati et al. proposed scanning 14 lung zones and assigning a score between zero to three points per zone; the total number of points would correlate with the severity of COVID-19 pneumonia 21. The same group analyzed protocols with fewer lung zones (e.g., four to twelve zones), given that an abbreviated protocol could help reduce exposure time of the scanner with infectious patients; the study concluded that a 12-zone protocol (including 4 posterior zones) was optimal to assess COVID-19 severity 22. The same group has validated their protocol for prognosis of COVID-19 pneumonia 23, but to our knowledge none has attempted to validate it for diagnosis of COVID-19 pneumonia (particularly for with a 12-zone protocol following Soldati’s score). Notably, there are several valuable studies suggesting the utility of POCUS for possible diagnosis of COVID-19 24, 25, 26, 27, 28, 29. However, these studies have limitations which hinder their generalizability, such as using a single operator, relying on the scanner’s overall subjective gestalt for COVID-19 pneumonia, not comparing to CT scan, or excluding patients with heart failure (which may have similar POCUS findings as COVID-19 pneumonia). In addition, few studies have gathered all data using handheld, ultra-portable devices.

Our aim was to validate a 12-zone protocol following Soldati et al. with a handheld device, assessing the diagnostic accuracy of lung POCUS performed with a handheld ultrasound machine to detect either (1) a high-suspicion (“typical”) pattern for COVID-19 on CT scan or (2) a positive COVID-19 NAT. We hypothesized that handheld lung POCUS has superior sensitivity and specificity compared to CXR for both outcomes. 

## Methods

### Study design and setting

This prospective cohort study took place in a 1,000-bed quaternary care hospital in the Northeastern U.S., from April to July 2020 (the first wave of the pandemic). We included a convenience sample of adult patients who were admitted to either the medical ward or intensive care unit (ICU). We calculated that we would need to recruit 70 patients, estimating a prevalence of 50% of cases with typical CT pattern (see Supplementary Material). A query of the electronic medical record system, roughly twice a week based on scanner availability, identified patients who had either confirmed or suspected COVID-19 infection (i.e., “PUI” status and awaiting further testing) and who had completed or were planned to complete a chest CT within 24 hours of the POCUS scan. Patients with a history of interstitial lung disease (ILD) were excluded, given these patients are relatively rare, typically know their underlying diagnosis, and at baseline will have quite abnormal lung POCUS scans. Otherwise, we attempted to recruit all consecutive patients. The protocol was approved by the local Partners Healthcare Institutional Review Board.

### Selection of participants, interventions, and measurements

**Figure 2:**
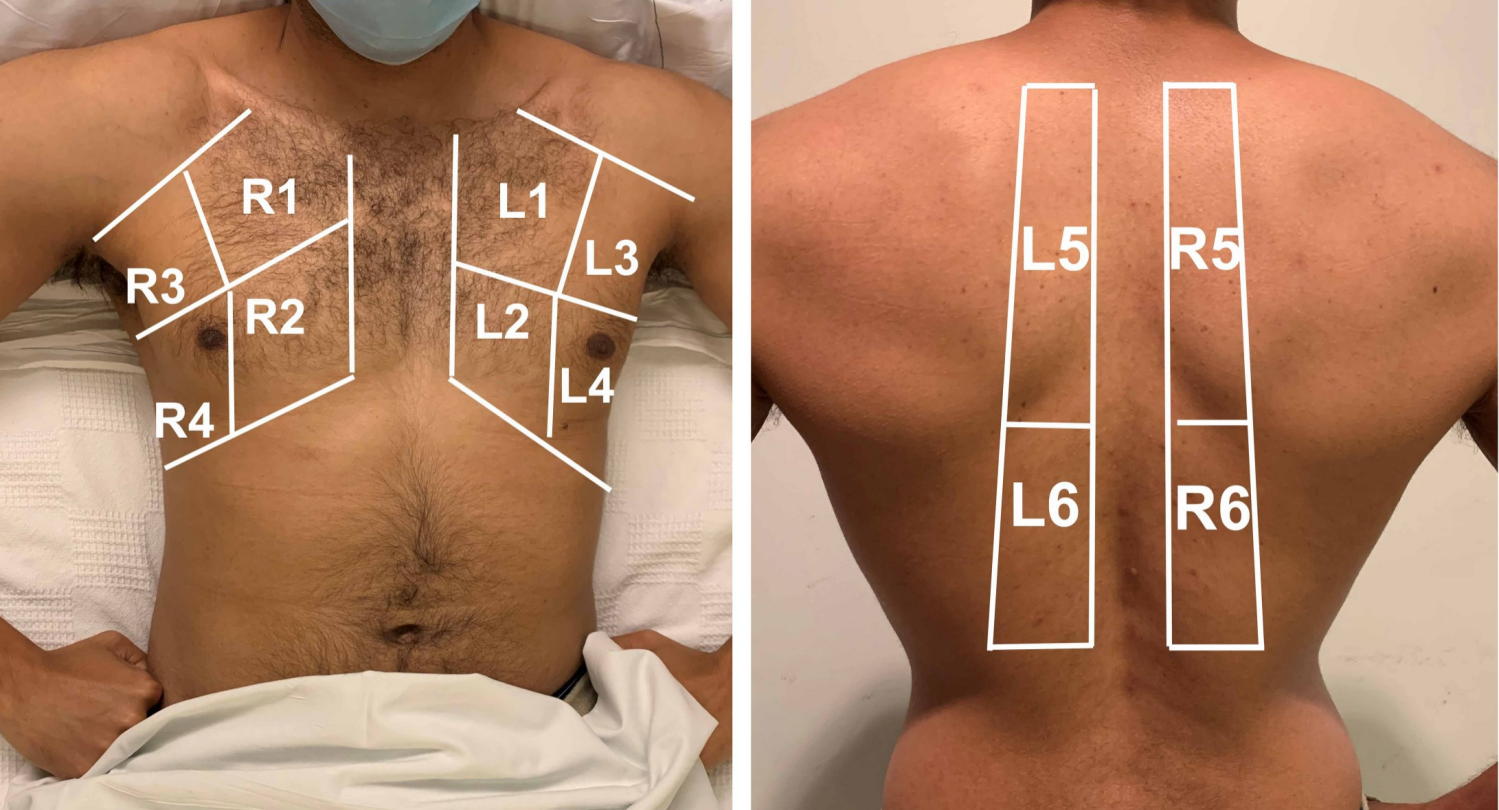
Lung Ultrasound Zones. Anterior lung zones (R1, R2, L1, L2) were scanned along the mid-clavicular line; lateral zones (R3, R3, L3, L4) were scanned along the mid-axillary line; the fourth rib separated the upper and lower anterior-lateral zones. Posterior lung zones (R5, R6, L5, L6) were scanned between the spine and the scapula; upper and lower posterior zones were separated by the inferior border of the scapula. Pictured is one of our physicians.

After obtaining assent from the treating team and verbal consent from the patient or proxy, a research physician scanned 12 lung zones (Figure 2), similar to prior protocols 24. The patient wore a surgical mask, and the scanning physician wore hospital-recommended personal protective equipment (N-95 respirator, eye protection, gown, and gloves) (Appendix Figure S1). The probe was held longitudinally and perpendicular to the ribs to obtain the “bat sign” view 25. Scanning physicians attempted to capture at least two intercostal spaces for each zone (and at least three intercostal spaces for each of the four posterior zones). For posterior lung zones, patients either sat upright or lay in lateral decubitus position. Scanning was completed using a handheld Butterfly iQ ultrasound machine (Butterfly Network, Inc., Guilford, CT) connected to an iPhone (Apple Inc., Cupertino, CA). The lung preset was used for all zones; a second clip was obtained using the abdominal preset for zones R4 and L4 (to investigate for pleural effusion). Single-use gel packets were used for ultrasound gel, to avoid cross-contamination. After scanning, machines were sanitized with hospital-approved disinfectant wipes. Scanning physicians included four internal medicine and two emergency medicine physicians, all of whom had completed formal POCUS training, including a minimum of 25 lung scans reviewed by a POCUS expert. All scanners were blinded to CT results, though not to NAT results (positive results are prominently displayed in the chart), at the time of scanning. Patient demographics, vital signs, amount of supplemental oxygen, and lab values were recorded by non-scanning research staff.

POCUS clips were reviewed by two Emergency Ultrasound Fellowship-trained physicians, who were blinded to CT and NAT results. Scans were scored along numerous criteria, including categories of B-lines, pleural line irregularity, and consolidation, selecting the most severe pathology in each of these 3 categories for each lung zone (see Appendix Figure S2). Examples are pictured (Figure 1). Similarly, CTs and CXRs were reviewed by two board-certified radiologists specialized in thoracic imaging and blinded to clinical and POCUS information. Radiologists were blinded to CT images when reading CXRs, and vice versa. Radiologists gave each CT and CXR a COVID-19 suspicion grade, following consensus criteria (namely, RSNA criteria for CT scans and British Society of Thoracic Imaging (BSTI) criteria for CXRs): namely, (1) typical/high suspicion, (2) indeterminate, (3) atypical/low suspicion, or (4) negative 14, 26. A third radiologist provided an interpretation in the case of discordant interpretations (as a tiebreaker). For both CT and CXR, the mode of the three interpretations was considered the consensus. For cases in which the third reader assigned a grade different from the primary two readers, the grade corresponding to the value closest to the median of the three observer grades was used as the consensus.

### Analysis

For all imaging modalities, we calculated a Cohen’s kappa to compare the interrater reliability between the first two readers (not including the “tiebreaker”). An ordinal scale was used for CT, CXR, and POCUS interpretations. For POCUS scans, we recorded the duration of time spent scanning (from the start of the first clip to the final clip).

We assessed the accuracy of the 12-zone POCUS protocol to detect either (1) typical CT pattern or (2) positive NAT (by the time of discharge). The Soldati protocol assigned a score between zero to three for each of the lung zones (thus, for our 12 zones, the maximum score for a patient was 36) 21. 

To calculate individual test characteristics (sensitivity, specificity, positive and negative predictive value) for our simplified POCUS score, we created two score cutoffs. These cutoffs allowed us to create low, intermediate, and high COVID-suspicion categories from the ordinal POCUS score (i.e., 0-36 points); these categories would mirror the RSNA CT and BSTI CXR categories. Data analysis was performed in Python 3.8.3 (Python Software Foundation, Beaverton, OR) 27. 

Receiver operating characteristic (ROC) curves were created to compare the performance of POCUS and CXR for our first outcome: a typical pattern for COVID-19 on CT. Additional ROC curves compared Soldati score, CXR, and CT against our second outcome: positive NAT. Areas under the curve (AUCs) were generated with the algorithm suggested by DeLong, DeLong, and Clark-Pearson 28. AUCs were compared with the test of equality of receiver operating areas in STATA IC 14.2 (Stata Corp LLC, College Station, TX).

## Results

Ninety patients were initially screened, and 79 were scanned and included in the final analysis. The remaining eleven were excluded based on lack of consent, presence of exclusion criteria (namely ILD), or CT scan not completed (Appendix Figure S3). Patient characteristics at the time of scan are given in Table 1. Most subjects were male (67.1%), of advanced age (mean age 62.5 years), overweight (mean BMI 27.0 kg/m^2^), and with elevated inflammatory markers. 

**Table 1:** Patient characteristics at the time of POCUS scan

-	**All** **n=79**	**COVID-19** **NAT positive** **n=21 (26.6%)**	**COVID-19** **NAT negativen=58 (73.4%)**	**p-value**
Age (years); mean (95% CI)	62.5 (58.6-66.4)	58.6 (50.0-67.2)	63.9 (59.7-68.2)	0.231
Gender female; %	32.9%	28.6%	34.5%	0.788
Scanned in ICU; %	5.1%	14.3%	1.7%	0.055
Body mass index (kg/m^2^); median (95% CI)	26 (25-27.7)	29 (25.5-31)	25.5 (24-26.9)	0.125
History of CHF; %	21.5%	14.3%	24.1%	0.537
Supplemental O_2_ (L/min)^1^; mean (95% CI)	2.7 (2.2-3.3)	2.6 (1.2-3.9)	2.8 (2.3-3.4)	0.670
Absolute lymphocyte count (per µL); median (95% CI)	1,125 (972-1,360)	1,280 (814-1,545)	1,110 (943-1,447)	0.710
Ferritin (ng/mL); median (95% CI)	353 (230-510)	691 (426-870)	220 (142-353)	0.001
CRP (mg/L); median (95% CI)	62.1 (38.5-84.8)	71.8 (48.7-132.8)	52.8 (11.6-85.9)	0.145
LDH (U/L); median (95% CI)	258 (226-326)	327 (266-394)	224 (180-258)	0.003
D-dimer (ng/mL); median (95% CI)	1,341 (1,066-1,577)	1,431 (891-2,435)	1,314 (1,010-1,573)	0.738
NT-proBNP (pg/mL); median (95% CI)	548 (307-2,044)	307 (34-1,843)	600 (322-2,844)	0.139
In-hospital death; %	7.6%	14.3%	5.2%	0.333
Discharged to hospice; %	1.3%	0.0%	1.7%	1.000
^1 ^Includes only 28 patients on nasal cannula. Abbreviations: NAT, nucleic acid test; CI; confidence interval; CHF, congestive heart failure; ICU, intensive care unit; CRP, C-reactive protein; LDH, Lactate dehydrogenase; NT pro-BNP, N-terminal pro B-type natriuretic peptide.				

Overall, 26.6% (21/79) of patients were NAT positive. All tested positive on their initial NAT. No patient who had an initial negative NAT tested subsequently tested positive during the study period. For CT scan, patients received the following RSNA grades (consensus interpretation): 18.9% (15/79) negative, 27.8% atypical/low (22/79), 21.5% indeterminate (17/79), and 31.6% typical (25/79). Fifteen patients (18.9%) had both positive NAT and a typical CT pattern. Seventy-five patients (94.9%) had CXRs completed; of these, the radiology consensus was 25.3% (19/75) negative, 24.0% (18/75) atypical/low, 29.3% indeterminate (22/75), and 21.3% (16/75) typical for COVID-19. For POCUS scan, 20.2% (16/79) of patients were categorized as low-risk, 53.2% (42/79) were intermediate, and 26.6% (21/79) were high-risk (based on scoring system, below). Interrater reliability between the two readers for each of the imaging modalities was as follows: κ = 0.822 for CT scan, κ = 0.559 for CXR, κ = 0.704 for POCUS. The median time between POCUS exam and CT scan completion was 13 hours (95% confidence interval [CI]: 11.1, 16.8). For ten patients, >24 hours elapsed between scans, typically from delays in obtaining CT scan after initial order. The POCUS exam took a median of 10 (95% CI: 9.4; 10.8) minutes. There was no statistically significant difference in the POCUS scan time between NAT-positive and negative patients (p=0.845).

**Figure 3:**
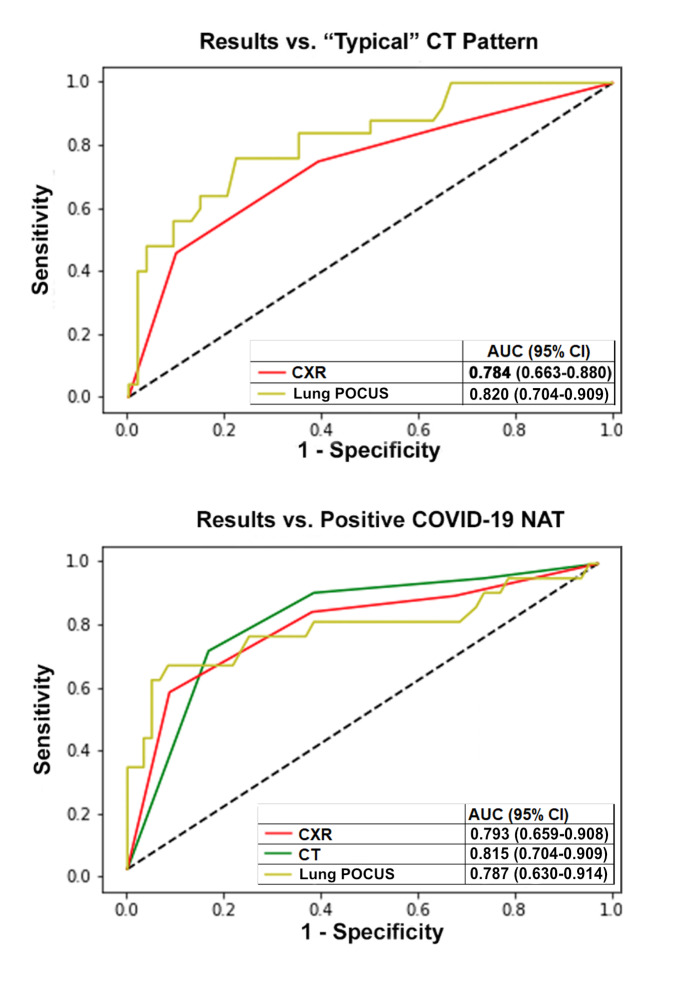
Receiver operating characteristic curves. Abbreviations: AUC, area under the curve; CI, confidence interval; CT, computed tomography; CXR, chest X-ray; NAT, nucleic acid test

The ROC curves with corresponding AUCs for CXR, CT, and POCUS are given in Figure 3. The AUC for POCUS was numerically higher than the AUC for CXR for the outcome of typical pattern on CT scan, but this difference did not reach statistical significance (p = 0.1201). To mirror the consensus categories used for CT and CXR, we created two cutoffs for the POCUS protocol, to divide patients into low, intermediate, and high suspicion for COVID. We set low suspicion at 6 or fewer points; intermediate at >6 and <24 points, and high suspicion at 24 or greater points. These cutoffs were chosen as practical method so that this protocol could be used to “rule-in” and “rule-out”; namely, to maximize sensitivity and NPV below the lower cutoff and maximize specificity and PPV above the higher cutoff--while also including a sizeable number of patients within each category. Operating characteristics are summarized in Table 2. The entire protocol is delineated in Table 3. 

**Table 2:** Test characteristics of POCUS protocol

-	**Sensitivity**	**Specificity**	**PPV**	**NPV**
**All Patients (n=79)**				
**For “Typical” CT Pattern:**				
Score ≥24	0.56	0.89	0.70	**0.81**
Score ≥6	1.00	0.30	0.40	**1.00**
**For Positive NAT:**				
Score ≥24	0.67	0.90	0.70	**0.88**
Score ≥6	0.90	0.24	0.30	**0.88**
Abbreviations: CT, computed tomography; NAT, nucleic acid test; NPV, negative predictive value; PPV, positive predictive value.				

**Table 3:** POCUS protocol adapted from Soldati, et al.

-Scan 12 zones with probe perpendicular to ribs, recording at least 2 intercostal spaces per zone (and 3 intercostal spaces per posterior zone) -Scoring: select the highest-scoring pathology for each zone. Points for the 12 zones are added to create a total score for each patient (maximum score = 36).	
**Score per zone:**	
**Finding**	**Points**
No B-lines or pleural line breaks	0
Any B-lines	1
Broken/irregular pleural line, or small (<1cm) subpleural consolidation	2
Light-beam artifact (confluent B-lines), or large subpleural consolidation, or hepatization	3
**Interpretation:**	
**Score**	**Interpretation**
< 6	Low suspicion for COVID-19
≥ 6 and < 24	Intermediate suspicion for COVID-19
≥ 24	High suspicion for COVID-19

## Discussion

A 12-zone lung POCUS protocol adapted from Soldati et al. and using handheld devices had reasonable diagnostic performance to detect either a typical CT pattern or a positive NAT for COVID-19. For patients with a higher POCUS score (high risk of COVID-19), the specificity was approximately 90% for detecting either typical CT pattern or positive NAT. For patients above the lower cut-off point, the sensitivities and negative predictive values for typical CT pattern and for positive NAT were all quite high (88-100%). There was no statistically significant difference in the diagnostic accuracy among POCUS, CT, and CXR. Thus, arguably patients with a high POCUS score could be considered high risk for having COVID-19, and further imaging with a CT might be avoided. Similarly, one might extrapolate that patients below the low cut-off could be considered to have a low risk for COVID-19 pneumonia (and might be able to forgo a CT scan that might be ordered to check for typical signs of COVID-19 pneumonia). 

The study results add to the literature that POCUS can assist in the diagnosis or risk stratification of PUIs for COVID-19 29, 30, 31, 32, 33. Namely, handheld lung POCUS may be a helpful adjunct to history and risk factors both to “rule in” or “rule out” COVID-19 pneumonia. Certainly, some of the more typical POCUS findings of COVID-19 can also be seen in other pulmonary conditions; but the same is true of typical CXR findings. The interrater reliability for readers for the POCUS score was overall good, notably higher than CXR (κ = 0.704 vs. κ = 0.559). Handheld POCUS, as used in this study, is relatively inexpensive and easy to disinfect, and may be particularly helpful in resource-limited settings, where access to CT or NAT may be limited or results may be delayed. In addition, the vast majority of scans were completed by internists with extra training in POCUS: notably, internists represent the largest medical specialty group in the United States 34. Finally, lung POCUS is rapid. Our 12-zone POCUS mean scanning time was ten minutes. However, this is not an insignificant amount of exposure time with an infectious patient, and it is a limitation of using POCUS. We plan to perform a subgroup analysis to assess whether a protocol with fewer lung zones would still perform well.

Our study is subject to several limitations. First, this was a single-center study, using a convenience sample of inpatients based on scanning physician availability. However, there have been relatively few prospective studies to date, particularly multi-center studies. Further validation studies performed at other centers are warranted and welcome. Second, this study was completed in 2020 in an earlier wave of the COVID-19 pandemic; newer variants seem to be causing severe disease and viral pneumonia less frequently 35. However, imaging is still useful and de facto being used. The presence of typical findings for COVID-19 pneumonia (whether on CT scan, or a high lung POCUS score) should still heighten concern for infection or complications, and some quaternary centers (including ours) continue to use chest imaging (CXR and CT) as part of the workflow to rule in and out COVID-19 infection 11. Whether the test characteristics described herein apply to current variants, vaccinated patients, and generally lower prevalence and disease severity is questionable and should be explored in newer cohorts. Third, our study examined only inpatients (mostly from the ward, with a small number of ICU patients); it is unknown whether this scanning protocol would yield similar results for outpatients or ED patients. Fourth, our sample size had a relatively narrow BMI range. Fifth, the relatively high positivity rate (26.6%) could lead to a spectrum effect. However, this incidence is lower than some other studies, and our sample did include many patients with low-suspicion CXR and CT results. Sixth, although scanning physicians were blinded to CT results, they were not blinded to NAT status (positive COVID status is displayed prominently in our hospital electronic record); it is possible that sampling bias of the lung zones was introduced. However, COVID-positive and PUI patients had similar POCUS scan times. Seventh, there is certainly a degree of subjectivity in interpreting different POCUS findings as described in the protocol from Soldati et al. (or any lung protocol). Our interrater reliability for POCUS readers was excellent, but the subjectivity could make the protocol less generalizable worldwide. Finally, none of the patients with an initial negative NAT subsequently had a positive NAT. However, this was an inpatient study, and initial NAT is always performed in the Emergency Department in our institution. Incidence of positive NAT after initial negative NAT is very low 2. Thus, it would be extremely challenging to capture an appreciable number of NAT-positive patients if we had limited our study to only those patients with an initial negative NAT. 

## Conclusions

Handheld lung POCUS could be a valuable tool in assessing the likelihood of COVID-19 pneumonia. A 12-zone protocol following Soldati et al. and using handheld devices performed well to detect patients with either a high-suspicion CT pattern for COVID-19 or a positive NAT. In patients under investigation for COVID-19, particularly in low-resource settings, a lung POCUS exam with an inexpensive, handheld machine could potentially replace other chest imaging as a helpful adjunct for clinicians to decide whether a diagnosis of COVID-19 pneumonia should be further entertained or no. 

## Ethics approval and consent to participate

The protocol was approved by our local Partners Healthcare (MassGeneral Brigham) Institutional Review Board. Verbal consent was obtained for every patient (via patient or patient proxy) to be scanned and participate in the study.

## Availability of data and material

The datasets used and analyzed during the current study are available from the corresponding author on reasonable request.

## Competing interests

The authors declare that they have no competing interests.

## Funding

The authors did not receive any funding for this research.

## Supplementary Material

Appendix Supplementary Figures and Plan for Statistical Methods and Sample Size Calculation from the Study Protocol.

## References

[R210597029282236] Kortela E, Kirjavainen V, Ahava M J, Jokiranta S T, But A, Lindahl A (2021). Real-life clinical sensitivity of SARS-CoV-2 RT-PCR test in symptomatic patients. PLoS One.

[R210597029282223] Dugdale C M, Anahtar M N, Chiosi J J, Lazarus J E, Mccluskey S M, Ciaranello A L (2021). Clinical, laboratory, and radiologic characteristics of patients with initial false-negative SARS-CoV-2 nucleic acid amplification test results. Open Forum Infect Dis.

[R210597029282220] Patrucco F, Carriero A, Falaschi Z, Pasché A, Gavelli F, Airoldi C (2021). COVID-19 diagnosis in case of two negative nasopharyngeal swabs: association between chest CT and bronchoalveolar lavage results. Radiology.

[R210597029282205] Long D R, Gombar S, Hogan C A, Greninger A L, Shah Vor, Bryson-Cahn C (2020). Occurrence and timing of subsequent SARS-CoV-2 RT-PCR positivity among initially negative patients. Clinical Infectious Diseases.

[R210597029282221] Dinnes J, Deeks J J, Berhane S, Taylor M, Adriano A, Davenport C (2021). point-of-care antigen and molecular-based tests for diagnosis of SARS-CoV-2 infection. Cochrane Database Syst Rev.

[R210597029282230] Fang Y, Zhang H, Xie J, Lin M, Ying L, Pang P (2020). Sensitivity of Chest CT for COVID-19: Comparison to RT-PCR. Radiology.

[R210597029282204] Ai T, Yang Z, Hou H, Zhan C, Chen C, Lv W (2020). Correlation of Chest CT and RT-PCR Testing in Coronavirus Disease 2019 (COVID-19) in China: A Report of 1014 Cases. Radiology.

[R210597029282235] Schalekamp S, Bleeker-Rovers C P, Beenen Lfm, Ufford Hme Quarles Van, Gietema H A, Stöger J L (2021). Chest CT in the Emergency Department for Diagnosis of COVID-19 Pneumonia: Dutch Experience. Radiology.

[R210597029282232] Som A, Lang M, Yeung T, Carey D, Garrana S, Mendoza D P (2020). Implementation of the Radiological Society of North America Expert Consensus Guidelines on Reporting Chest CT Findings Related to COVID-19: A Multireader Performance Study. Radiology: Cardiothoracic Imaging.

[R210597029282206] Schalekamp S, Bleeker-Rovers C P, Beenen Lfm, Hmeqv Ufford, Gietema H A, Stöger J L (2021). Chest CT in the Emergency Department for Diagnosis of COVID-19 Pneumonia: Dutch Experience. Radiology.

[R210597029282237] Dugdale C M, Rubins D M, Lee H, Mccluskey S M, Ryan E T, Kotton C N (2021). Coronavirus Disease 2019 (COVID-19) Diagnostic Clinical Decision Support: A Pre-Post Implementation Study of CORAL (COvid Risk cALculator). Clinical Infectious Diseases.

[R210597029282208] Rubin G D, Ryerson C J, Haramati L B, Sverzellati N, Kanne J P, Raoof S (2020). The Role of Chest Imaging in Patient Management during the COVID-19 Pandemic: A Multinational Consensus Statement from the Fleischner Society. Radiology.

[R210597029282231] Akl E A, Blažžić I, Yaacoub S, Frija G, Chou R, Appiah J A (2021). Use of Chest Imaging in the Diagnosis and Management of COVID-19: A WHO Rapid Advice Guide. Radiology.

[R210597029282207] Simpson S, Kay F U, Abbara S, Bhalla S, Chung J H, Chung M (2020). Radiological society of north America expert consensus document on reporting chest CT findings related to COVID-19: endorsed by the society of thoracic Radiology, the American college of Radiology, and RSNA. Radiology: Cardiothoracic Imaging.

[R210597029282217] Stephanie S, Shum T, Cleveland H, Challa S R, Herring A, Jacobson F L (2020). Determinants of chest x-ray sensitivity for covid-19: A multi-institutional study in the united states. Radiology: Cardiothoracic Imaging.

[R210597029282234] Little B P (2021). False-Negative Nasopharyngeal Swabs and Positive Bronchoalveolar Lavage: Implications for Chest CT in Diagnosis of COVID-19 Pneumonia. Radiology.

[R210597029282226] Volpicelli G, Gargani L (2020). Sonographic signs and patterns of COVID-19 pneumonia. The Ultrasound Journal.

[R210597029282214] Huang Y, Wang S, Liu Y, Zhang Y, Zheng C, Zheng Y (2020). A preliminary study on the ultrasonic manifestations of peripulmonary lesions of non-critical novel coronavirus pneumonia (COVID-19). https://ssrn.com/abstract=3544750.

[R210597029282213] Smith M J, Hayward S A, Innes S M, Miller Asc (2020). Point-of-care lung ultrasound in patients with COVID-19-a narrative review. Anaesthesia.

[R210597029282210] Peng Q-Y, Wang X-T, Zhang L-N, G Chinese Critical Care Ultrasound Study (2020). Chinese Critical Care Ultrasound Study G. Findings of lung ultrasonography of novel corona virus pneumonia during the 2019-2020 epidemic. Intensive care medicine.

[R210597029282233] Soldati G, Smargiassi A, Inchingolo R, Buonsenso D, Perrone T, Briganti D F (2020). Proposal for International Standardization of the Use of Lung Ultrasound for Patients With COVID-19: A Simple, Quantitative, Reproducible Method. Journal of Ultrasound in Medicine.

[R210597029282227] Mento F, Perrone T, Macioce V N, Tursi F, Buonsenso D, Torri E (2021). On the Impact of Different Lung Ultrasound Imaging Protocols in the Evaluation of Patients Affected by Coronavirus Disease 2019: How Many Acquisitions Are Needed?. J Ultrasound Med.

[R210597029282225] Soldati G, Smargiassi A, Perrone T, Torri E, Mento F, Demi L (2021). There is a Validated Acquisition Protocol for Lung Ultrasonography in COVID-19 Pneumonia. J Ultrasound Med.

[R210597029282215] Haak S L, Renken I J E, Jager L C, Lameijer H, Kolk B Y M van der (2021). Diagnostic accuracy of point-of-care lung ultrasound in COVID-19. Emergency Medicine Journal.

[R210597029282216] Lichtenstein D A (2014). Lung ultrasound in the critically ill. Annals of Intensive Care.

[R210597029282229] Hare S S, Tavare A N, Dattani V, Musaddaq B, Beal I, Cleverley J (2020). Validation of the British Society of Thoracic Imaging guidelines for COVID-19 chest radiograph reporting. Clin Radiol.

[R210597029282211] McKinney W (2010). Data structures for statistical computing in python.

[R210597029282238] DeLong E R, DeLong D M, Clarke-Pearson D L (1988). Comparing the areas under two or more correlated receiver operating characteristic curves: a nonparametric approach. Biometrics.

[R210597029282212] Pivetta E, Goffi A, Tizzani M, Locatelli S M, Porrino G, Losano I (2021). Lung Ultrasonography for the Diagnosis of SARS-CoV-2 Pneumonia in the Emergency Department. Ann emerg med.

[R210597029282218] Lieveld A W E, Kok B, Schuit F H, Azijli K, Heijmans J, Laarhoven A van (2020). Diagnosing COVID-19 pneumonia in a pandemic setting: Lung Ultrasound versus CT (LUVCT)-a multicentre, prospective, observational study. ERJ Open Research.

[R210597029282209] Volpicelli G, Gargani L, Perlini S, Spinelli S, Barbieri G, Lanotte A (2021). Lung ultrasound for the early diagnosis of COVID-19 pneumonia: an international multicenter study. Intensive care medicine.

[R210597029282222] Peixoto A O, Costa R M, Uzun R, Fraga Ama, Ribeiro J D, Marson F A L (2021). Applicability of lung ultrasound in COVID-19 diagnosis and evaluation of the disease progression: A systematic review. Pulmonology.

[R210597029282228] Haak S L, Renken Ije, Jager L C, Lameijer H, Kolk B B Y M van der (2021). Diagnostic accuracy of point-of-care lung ultrasound in COVID-19. Emergency Medicine Journal.

[R210597029282224] (2019). Active Physicians in the Largest Specialties. Association of American Medical Colleges.

[R210597029282219] Nyberg T, Ferguson N M, Nash S G (2022). Comparative analysis of the risks of hospitalisation and death associated with SARS-CoV-2 omicron (B.1.1.529) and delta (B.1.617.2) variants in England: a cohort study. Lancet.

