# Supplementary material for: Handheld Lung Ultrasound to Detect COVID-19 Pneumonia in Inpatients: A Prospective Cohort Study
Source: POCUS J. 2023 Nov 27;8(2):175–83. doi: 10.24908/pocus.v8i2.16484 (PMC10721309; doi:10.24908/pocus.v8i2.16484)
Supplement: Appendix [file pocusj-08-16484-s001.pdf]

Handheld lung ultrasound to detect COVID-19 pneumonia in inpatients: A validation study

APPENDIX

Supplemental Figure 1: Scanner and patient positioning and use of personal protective equipment.

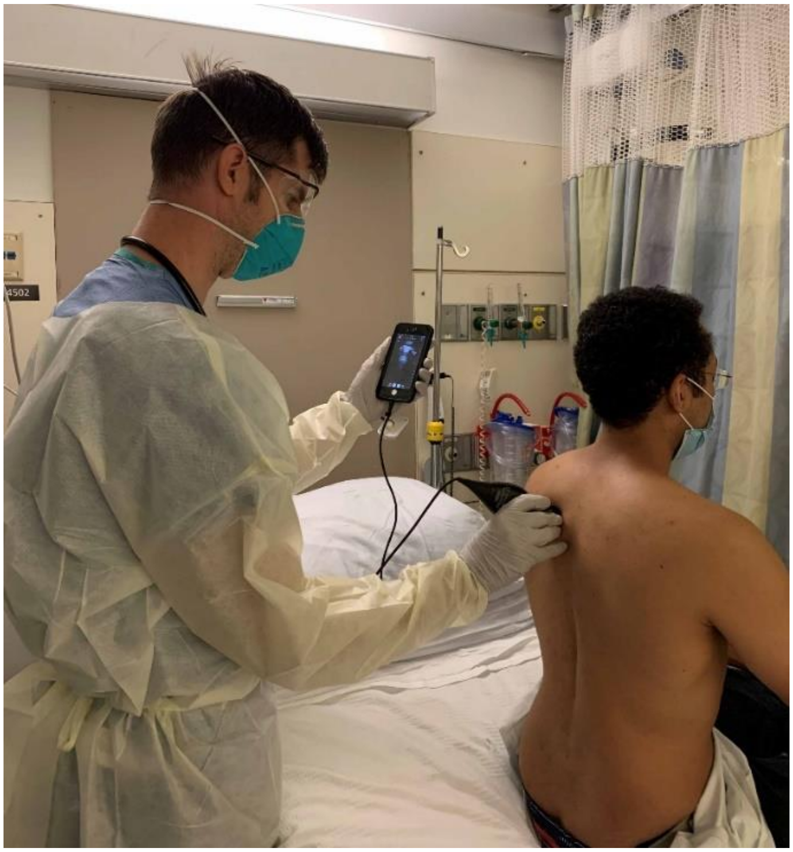

Legend: Pictured is a scanning physician, with another physician acting as model.

Supplemental Figure 2: POCUS Reader Scoring Form

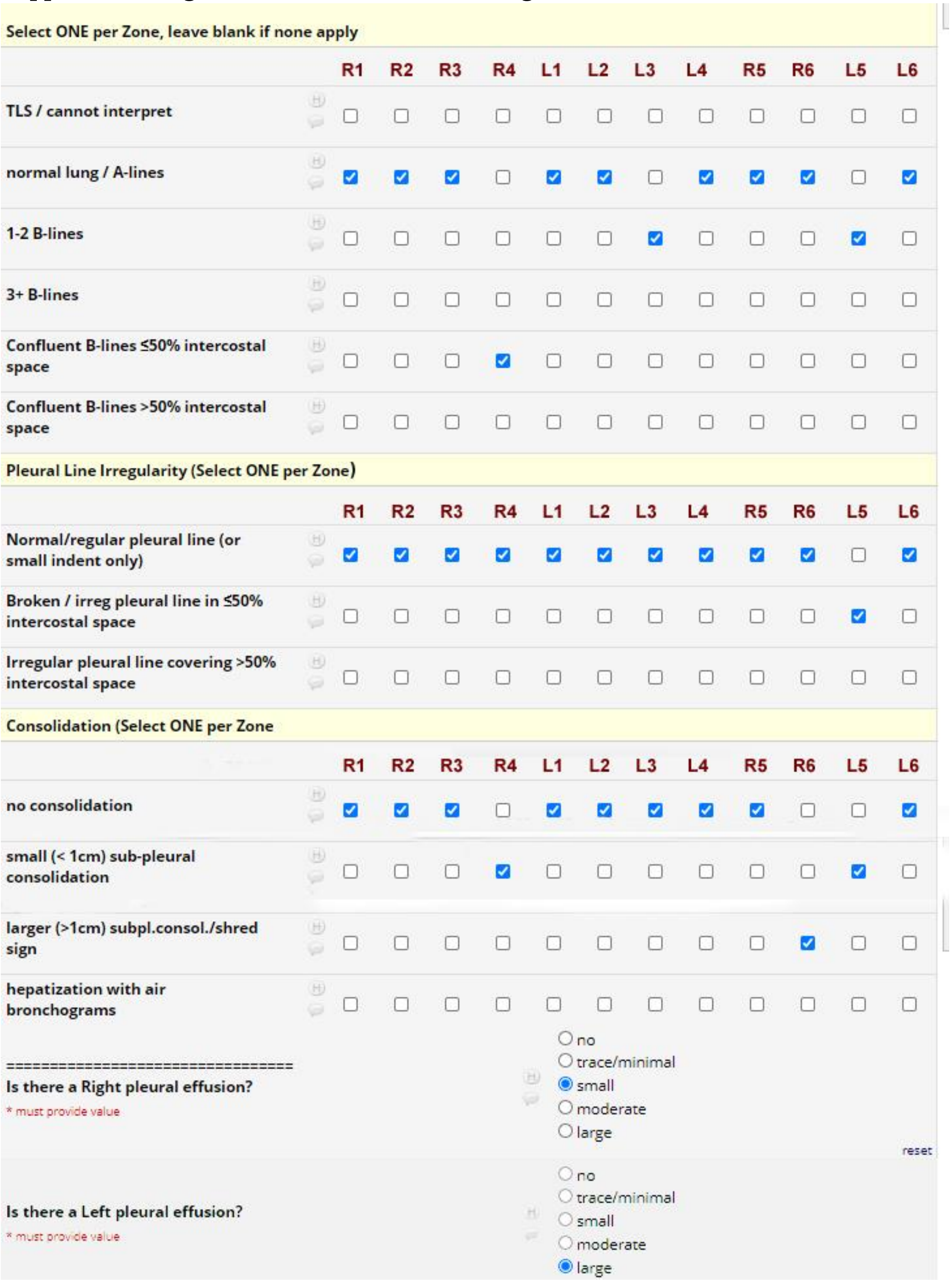

Select ONE per Zone, leave blank if none apply	R1	R2	R3	R4	L1	L2	L3	L4	R5	R6	L5	L6
TLS / cannot interpret	☐	☐	☐	☐	☐	☐	☐	☐	☐	☐	☐	☐
normal lung / A-lines	☑	☑	☑	☐	☑	☑	☐	☑	☑	☑	☐	☑
1-2 B-lines	☐	☐	☐	☐	☐	☐	☑	☐	☐	☐	☑	☐
3+ B-lines	☐	☐	☐	☐	☐	☐	☐	☐	☐	☐	☐	☐
Confluent B-lines ≤50% intercostal space	☐	☐	☐	☑	☐	☐	☐	☐	☐	☐	☐	☐
Confluent B-lines >50% intercostal space	☐	☐	☐	☐	☐	☐	☐	☐	☐	☐	☐	☐

Pleural Line Irregularity (Select ONE per Zone)	R1	R2	R3	R4	L1	L2	L3	L4	R5	R6	L5	L6
Normal/regular pleural line (or small indent only)	☑	☑	☑	☑	☑	☑	☑	☑	☑	☑	☐	☑
Broken / irreg pleural line in ≤50% intercostal space	☐	☐	☐	☐	☐	☐	☐	☐	☐	☐	☑	☐
Irregular pleural line covering >50% intercostal space	☐	☐	☐	☐	☐	☐	☐	☐	☐	☐	☐	☐

Consolidation (Select ONE per Zone	R1	R2	R3	R4	L1	L2	L3	L4	R5	R6	L5	L6
no consolidation	☑	☑	☑	☐	☑	☑	☑	☑	☑	☐	☐	☑
small (< 1cm) sub-pleural consolidation	☐	☐	☐	☑	☐	☐	☐	☐	☐	☐	☑	☐
larger (>1cm) subpl.consol./shred sign	☐	☐	☐	☐	☐	☐	☐	☐	☐	☑	☐	☐
hepatization with air bronchograms	☐	☐	☐	☐	☐	☐	☐	☐	☐	☐	☐	☐

==================================

Is there a Right pleural effusion?
* must provide value

- ○ no
- ○ trace/minimal
- ◉ small
- ○ moderate
- ○ large

reset

Is there a Left pleural effusion?
* must provide value

- ○ no
- ○ trace/minimal
- ○ small
- ○ moderate
- ◉ large

Abbreviations: TLS, technically limited study; irreg, irregular; subpl.consol., subpleural consolidation

Supplemental Figure 3: Study Population

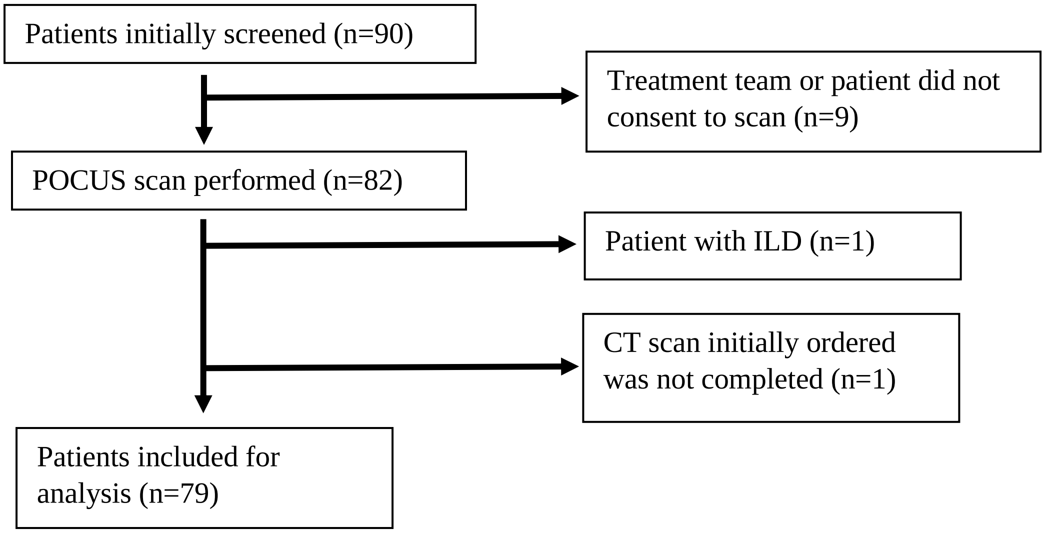

Plan for Statistical Methods and Sample Size Calculation from the Study Protocol

For the primary endpoint, sensitivity, our hypothesis is a sensitivity of 0.90 of POCUS compared to CT, with a variance of 0.1 and a precision of 0.1.

We will calculate sensitivity, specificity, accuracy, PPV, and NPV of POCUS compared CT based on the dichotomous variable "high suspicion for COVID-19" on CT and POCUS, respectively.

Inter-rater reliability (between POCUS and CT) will be calculated via Cohen's Kappa. We hypothesize a κ of 0.61 to 0.80, equivalent to substantial agreement between the two modalities.

We will also compare the inter-rater reliability between POCUS readers and between CT readers, respectively.

The ROC curve will be constructed via the different cut-off points for POCUS, and we will calculate the AUC for POCUS compared to CT. We will use the patient-level data for a non-parametric estimation of the Bamber and Hanley confidence intervals for the area under the ROC. We test our hypothesis of an AUC of 0.9 compared to the gold standard. Finally, we will adjust analyses for age and gender via a ROC regression. We will also conduct subgroup analyses in patients with a history of CHF and in patients with morbid obesity (BMI $\geq$ 40).

Assuming a 5% type I error rate and a 95% confidence level, we calculate a sample size of n=35 positive cases, i.e. patients with a CT scan with "high suspicion for COVID-19" via the formula

$$N = \frac{(Z_{\alpha/2})^2 \times Variance}{Precision^2}$$

Considering a prevalence of 50% of cases with a positive a CT scan with "high suspicion for COVID-19", this would require n=70 patients to be scanned via POCUS and CT scan. We would approach a n=78 patients, assuming a response rate of 90%.
